# Presence of Carpal Tunnel Syndrome and Its Impact on Clinical and Ultrasonographic Evaluations in Patients with Fibromyalgia

**DOI:** 10.5152/ArchRheumatol.2026.25157

**Published:** 2026-01-16

**Authors:** Selcuk Akkaya, Gonca Saglam Akkaya, Kaan Alisar, Dilek Cetinkaya Alisar, Serdar Karakullukcu

**Affiliations:** 1 Department of Radiology, Karadeniz Technical University, Trabzon, Türkiye; 2 Department of Physical Medicine and Rehabilitation, Karadeniz Technical University, Trabzon, Türkiye; 3 Department of Radiology, Erzurum Regional Training and Research Hospital, Erzurum, Türkiye; 4 Department of Physical Medicine and Rehabilitation, Erzurum Regional Training and Research Hospital, Erzurum, Türkiye; 5 Bayburt Community Health Center, Bayburt, Türkiye

**Keywords:** Carpal tunnel syndrome, fibromyalgia, musculoskeletal ultrasound

## Abstract

**Background/Aims:**

Carpal tunnel syndrome (CTS) may be overlooked in patients with fibromyalgia (FM) due to the high prevalence of paresthesia and hand pain. This study aimed to determine the presence of CTS in patients with FM compared to healthy controls and to identify potential associations between clinical parameters and ultrasound (US).

**Materials and Methods:**

This study included 40 patients with FM (80 wrists) and 36 healthy controls (72 wrists). The presence of paresthesia, sensory/motor deficits, and Tinel/Phalen test results was recorded. All participants underwent US to assess the median nerve cross-sectional area (CSA), distal/proximal ratio, and intraneural power Doppler signal (PDS). Electrodiagnostic studies were conducted in the presence of any CTS-related symptom. The Boston Carpal Tunnel Syndrome Questionnaire symptom severity (BCTQs) and functional status (BCTQf) scale, and the Fibromyalgia Impact Questionnaire (FIQ) were recorded for all FM patients.

**Results:**

Carpal tunnel syndrome was identified in 18 wrists (22.5%) of 14 patients (35%) in the FM group, whereas it was present in only 2 wrists (2.8%) of a single participant (2.8%) in the control group (*P* < .001). No statistically significant differences were observed in the median nerve CSA, distal/proximal ratio, and PDS between the groups (*P* = .727, *P* = .270, and *P* = .058, respectively). Median nerve CSA was moderately correlated with BCTQs, BCTQf, and FIQ (*r* = 0.557, *r* = 0.599, *r* = 0.553; all *P* < .001) in patients with FM. Median nerve CSA was greater in moderate (1.31 [1.30-1.47]) than mild CTS (1.13 [1.00-1.20]) (*P* < .001).

**Conclusion:**

Carpal tunnel syndrome appears to be a common clinical manifestation of FM. Ultrasonographic evaluation demonstrated significant correlations with clinical scales, highlighting its value as a complementary tool in evaluating CTS in FM.

**Main Points**

- Carpal tunnel syndrome (CTS) was significantly more prevalent in patients with FM compared to healthy controls.
- Fibromyalgia (FM) patients with CTS had worse clinical scale scores compared to those without CTS.
- Median nerve cross-sectional area and distal/proximal ratio were correlated with the Boston Carpal Tunnel Syndrome Questionnaire symptom severity, Boston Carpal Tunnel Syndrome Questionnaire functional status, and Fibromyalgia Impact Questionnaire in FM patients.

## Introduction

Fibromyalgia (FM) is a chronic pain disorder characterized by widespread musculoskeletal pain lasting at least 3 months.^1^^,^^2^ Beyond pain, patients frequently report persistent fatigue, sleep disturbances, cognitive dysfunction, and mood disturbances, including anxiety and depression.^3^^-^^5^ Many also experience additional somatic complaints, including stiffness, headaches, and gastrointestinal disturbances, reflecting the multisystem nature of the syndrome.^3^^,^^6^

The widespread pain characteristic of FM is often accompanied by an array of distinct sensory symptoms, including paresthesia (tingling or prickling sensations) and allodynia (pain from non-painful stimuli), which underscore the neurological complexity of the disorder.^7^^-^^9^ While FM is believed to involve dysregulation of central pain processing mechanisms, resulting in enhanced pain sensitivity and impaired inhibitory control, peripheral factors, including small fiber neuropathy and localized nerve compression, have also been implicated in the pathophysiology of sensory symptoms.^10^^,^^11^

Entrapment neuropathies, which occur when peripheral nerves are compressed at specific anatomical sites, represent a common cause of pain and neurological symptoms, with carpal tunnel syndrome (CTS) being the most prevalent among them.^12^ Carpal tunnel syndrome is of particular clinical and pathophysiological relevance in the context of FM for several key reasons. First, some studies have shown a significantly increased prevalence of CTS in individuals with FM compared to the general population, as high as 16% to over 20%.^13^^,14^ Second, the symptoms of CTS can overlap with the diffuse sensory disturbances characteristic of FM, creating a diagnostic challenge for clinicians.^13^^,^^15^ Furthermore, the focal nerve damage from CTS may act as a peripheral pain generator, potentially contributing to central sensitization and the overall pain burden in FM.^16^

Several studies have explored the relationship between FM and CTS, reporting inconsistent findings regarding the prevalence of CTS in FM patients. While several studies report a significantly higher prevalence of CTS in FM patients compared to the general population,^13^^,^^17^^-^^19^ other investigations suggest that the diagnostic confusion may often stem from the diffuse sensory symptoms of central sensitization mimicking neuropathic pain, rather than a true co-morbidity.^14^^,^^15^^,^^20^ Moreover, the relationship between structural nerve abnormalities, as detectable by ultrasound (US), and clinical symptom severity in this specific patient population remains poorly characterized. Therefore, the objective of this study is to assess the presence of CTS in patients with FM compared with a control group and to evaluate the association between US findings and clinical variables.

## Materials and Methods

### Study Population

This cross-sectional study enrolled 40 female patients with FM (80 wrists) and 36 healthy female controls (72 wrists), aged 18-75 years. Patients who presented to the Physical Medicine and Rehabilitation Department of Erzurum Regional Training and Research Hospital with FM symptoms between May 2021 and May 2022 and were diagnosed in accordance with the American College of Rheumatology 2016 FM diagnostic criteria^20^ were included in the study. The control group consisted of volunteers who were asymptomatic, exhibited no signs of musculoskeletal disorders, and had no symptoms or prior diagnosis of CTS.

Participants with polyneuropathy, radiculopathy, peripheral neuropathy, diabetes mellitus, history of a fracture, trauma, or surgery at the wrist level, and any inflammatory, neurological, autoimmune, endocrine, or renal diseases were excluded. Pregnant and lactating women were excluded from this study. Fibromyalgia patients who had carpal tunnel injection, physical therapy, and/or medical treatment in the last 6 months were not included in the study.

An ethical approval from the local ethics committee of Erzurum Regional Training and Research Hospital (Date: July 20, 2020; Number: 2020/14–160) was obtained, and all participants completed a written informed consent before the initiation of the study. Patient selection and recruitment were carried out in compliance with the Declaration of Helsinki.

### Demographic, Anthropometric, and Clinical Data

Demographic and anthropometric characteristics, including age, the dominant hand, and body mass index were recorded. Occupational factors, including repetitive hand movements, firmly grasping or pinching tools, putting the hands and wrists in uncomfortable positions, applying pressure directly over the carpal tunnel, and the utilization of vibrating hand-held tools, were also noted. Physical examination included the Tinel test, the Phalen test, and sensory and motor tests. Ultrasound assessments were performed for all the participants.

The diagnosis of CTS was based on the concurrence of both clinical and electrophysiological evidence. For a wrist to be included as a CTS case and proceed to electrodiagnostic studies (EDX), it was first required to exhibit at least 1 characteristic clinical symptom or sign (paresthesia/pain in the median nerve distribution, sensory/motor deficits, a positive Tinel test, or a positive Phalen test). Final CTS positivity was confirmed only when this clinical suspicion was supported by abnormal findings on EDX, which is the gold standard for objectively proving median nerve entrapment.^21^^,^^22^

The Boston Carpal Tunnel Syndrome Questionnaire (BCTSQ) and Fibromyalgia Impact Questionnaire (FIQ) were recorded for all FM patients. The BCTSQ assesses functional outcome (BCTQf) and severity of symptoms (BCTQs) unique to CTS. The severity of symptoms increased with higher scores. Eight functional activities were rated on a scale of 1-5 using the BCTQf, yielding a total BCTQf of 40. A higher score indicates lower functional capacity.^23^ The FIQ is a self-report instrument that evaluates the influence of FM symptoms on physical and mental health. The FIQ assesses physical disability, the number of days feeling well, work absenteeism and capacity, pain status, fatigue, stiffness, and symptoms of anxiety and depression. Each subscale was scored on a scale of 0-10, with the sum of these scores yielding a total of 0-100.^24^

### Electrodiagnostic Studies

Participants who had any CTS-related clinical symptom/sign underwent EDX using the Cadwell Sierra device (Cadwell Laboratories, Kennewick, WA, USA). A physical medicine and rehabilitation specialist (D.Ç.A), blinded to the clinical assessments and US measurements, evaluated the EDX results. The following standard upper normal limit values were employed in EDX studies: (i) 3.6 ms is the median nerve sensory distal latency and (ii) A sensory distal latency difference of less than 0.4 ms between the median and ulnar nerves. The median nerve distal motor latency measured 8 cm from the upper thenar muscle was 4.3 ms. Impairment in both distal motor delay and sensory nerve conduction was considered as moderate CTS, whereas cases that affected only sensory nerve conduction were considered as mild CTS. Severe CTS was defined as cases in which the motor delay was prolonged, and there was no sensory or, in certain cases, a motor response.^25^^,^^26^

### Ultrasonography

An experienced radiologist (K.A.) who was blinded to the clinical and EDX findings of the patients performed the sonographic evaluation of the distal forearm and wrist. A Toshiba Aplio 500 US (Toshiba Medical Systems, Tokyo, Japan) with a broadband 6-18 MHz linear transducer was used for the sonographic examinations. The participants were seated facing the sonographer with their hands and wrists resting on the examination table and fingers slightly in a semi-flexed position. The median nerve cross-sectional area (CSA) was evaluated distally at the pisiform bone level, using it as an anatomical reference point, and was measured by carefully delineating an uninterrupted line along the inner border of the hyperechogenic epineurial rim, thus encompassing the nerve fascicles. The proximal portion of the median nerve CSA was evaluated superior to the distal third of the pronator quadratus muscle. Each CSA measurement was taken 3 times, and the average values were utilized for subsequent analysis.^27^ The distal/proximal ratio was determined by dividing the median nerve’s CSA at the pisiform bone level by its proximal CSA. Intraneural power Doppler signal (PDS) was evaluated using a quantitative grading scale ranging from 0 to 3 (0 = normal, 1 = mild, 2 = moderate, 3 = marked), employing a method previously described by Filippucci et al.^28^

### Statistical Analysis

Sample size was determined using G*Power 3.1 (Heinrich Heine University of Düsseldorf; Düsseldorf, Germany). The calculation was based on detecting a difference in CTS prevalence between the FM and control groups, using an alpha of 0.05, a power of 80%, and an effect size of 0.7 derived from the study by Nacir et al.^14^ This yielded a minimum sample size of 70 participants (35 participants in each group).

SPSS version 23.0 (IBM SPSS Corp.; Armonk, NY, USA) was employed for data analysis. Categorical variables are summarized using frequencies and percentages, while continuous variables are described using means and SDs. The Kolmogorov–Smirnov test was utilized to assess the normality of distribution across groups. For comparing non-normally distributed data between patient and control groups, the Mann–Whitney *U*-test was applied. Qualitative data comparisons were performed using chi-square and Fisher’s exact tests. Correlation analysis was carried out using the Spearman test. The following guideline was used to interpret the correlation coefficients: correlation coefficients ranging from 0.3 to 0.5 suggest a mild positive linear association, those between 0.5 and 0.7 indicate a moderate positive linear correlation, values from 0.7 to 0.9 denote a strong positive linear relationship, and coefficients between 0.9 and 1 signify a very strong positive linear correlation.^29^ *P*-values ≤.05 were considered statistically significant.

## Results

No statistically significant difference was observed in terms of age between the FM group (43.5 [33.25-52]) and the control group (36.5 [31-41]) (*P* = .375). There was no statistically significant difference between the FM and control groups regarding body mass index (BMI), occupational risk factors, and hand dominance (*P* = .941, *P* > .99, *P* > .99, respectively) (Table 1).

The mean ± SD for the key median nerve conduction parameters in the CTS positive FM patients were as follows: motor distal latency was 3.11 ± 0.85 ms, sensory conduction velocity was 38.86 ± 5.25 m/s, sensory action potential amplitude was 44.51 ± 15.71 µV, and compound muscle action potential amplitude was 10.57 ± 2.64 µV.

The mean ± SD for the key median nerve conduction parameters in the CTS negative FM patients were as follows: motor distal latency was 2.44 ± 0.52 ms, sensory conduction velocity was 44.07 ± 6.47 m/s, sensory action potential amplitude was 47.50 ± 13.54 µV, and compound muscle action potential amplitude was 12.60 ± 2.66 µV.

Among the wrists of patients with FM, 12 (15%) demonstrated mild CTS, whereas 6 (7.5%) exhibited moderate CTS. Four FM patients and 1 control subject had bilateral CTS. No statistically significant differences were detected between the FM and control groups in terms of intraneural PDSs, median nerve CSA, and distal/proximal ratio (*P* = .058, *P* = .727, and *P* = .270, respectively) (Table 2).

The distal/proximal ratio of the median nerve was significantly higher in FM patients with CTS (1.66 [1.44-1.72]) compared to FM patients without CTS (0.85 [0.75-0.97]) (*P* = .001); however, no statistically significant difference was detected in the median nerve CSA between the 2 groups (8.2 [6.8-9.4] vs. 7.1 [6.2-8.0]; *P* = .141). Fibromyalgia patients with CTS demonstrated significantly higher BCTQs, BCTQf, and FIQ scores compared to FM patients without CTS (*P* < .001, *P* < .001, and *P* < .001, respectively) (Table 3).

Median nerve CSA was significantly greater in patients with moderate CTS (1.31 [1.30-1.47]) compared with those with mild CTS (1.13 [1.00-1.20]) (*P* < .001). However, no significant difference was observed between the mild and moderate CTS groups with respect to the distal/proximal ratio (1.68 [1.20-1.71] vs. 1.65 [1.52-1.73]; *P* = 1.000).

Median nerve CSA was moderately correlated with BCTQs, BCTQf, and FIQ scores in FM patients (*r* = 0.557, *r *= 0.599, and *r* = 0.553, respectively; all *P*-values < .001). A weak positive correlation was detected between distal/proximal ratio and BCTQs, BCTQf, and FIQ scores (*r* = 0.431, *P* < .001; *r* = 0.435, *P* < .001; and *r* = 0.336, *P* = .002, respectively) (Table 4).

## Discussion

A limited number of prospective studies with small sample sizes and retrospective studies, predominantly lacking clinical features, were conducted to determine the co-occurrence of FM and CTS, which present with similar symptoms and affect the same age and sex demographics. However, most previous investigations have evaluated CTS in patients with FM solely based on EDX findings, without incorporating complementary imaging methods such as US. In this study, 35% of FM patients and 2.8% of the control group had CTS. Additionally, symptom and disease severity were associated with both EDX and US findings of the median nerve in patients with FM.

An increasing number of studies have focused on the coexistence of CTS and FM; however, variable results have been reported. Caro et al^11^ reported CTS at a rate of 24% in patients with FM and 29% in matched control subjects, according to EDX, with no statistically significant difference. Sarmer et al^15^ included 50 patients with FM and 50 healthy individuals, and CTS was detected in 10% and 4% of the cases, respectively. However, the authors did not pair their data for BMI, and FM patients had higher rates of obesity than the others. In FM patients, higher BMI is already linked to worse functional status and symptom burden.^30^ Furthermore, imaging studies show that obesity correlates with both larger median nerve CSA and prolonged nerve conduction parameters.^31^^,^^32^ Given these potential confounders, adjusting for BMI in comparisons between FM and control groups may improve the accuracy of CTS-related assessments.

The study by Nacir et al^14^ reported a CTS prevalence of 20.6% in FM patients and 2.8% in controls, findings that are comparable to, but slightly lower than, those observed in the present cohort (35% and 2.8%, respectively). Several methodological and population differences may explain this variation. First, Nacir et al^14^ exclusively relied on EDX for CTS diagnosis, whereas the current study employed a combined clinical approach and EDX, which likely increased diagnostic sensitivity by identifying subclinical cases. Second, their study excluded individuals with obesity and other metabolic comorbidities, while the current cohort reflected a broader BMI range, allowing evaluation of its potential contribution to CTS risk. Third, differences in diagnostic criteria (1990 vs. 2016 American College of Rheumatology criteria for FM) may have also contributed to higher detection rates in this study. Additionally, the inclusion of validated clinical scales (BCTQs, BCTQf, and FIQ) enabled a more comprehensive assessment of the functional and symptomatic impact of CTS in FM.^14^

Electrodiagnostic studies testing provides a quantitative evaluation of the median nerve’s physiological function, whereas US examination of the wrist may demonstrate compression-linked edema and vascular differentiation in CTS, resulting in a larger median nerve CSA.^33^ Ultrasound has been demonstrated as a potential diagnostic tool for CTS. In contrast to the previous studies mentioned above, the current study investigated the participants using both EDX and US. A consistent, moderate correlation was found between the median nerve CSA and validated clinical scales in patients with FM. This finding aligns with the established role of CSA as a reliable sonographic biomarker of compressive neuropathy severity.^27^^,^^34^^,^^35^ On the other hand, some patients exhibiting typical clinical signs of CTS may have normal EDX results, yet sonographic irregularities might be detected. Borire et al^36^ evaluated sonographic parameters in wrists clinically diagnosed with CTS, categorizing them by EDX findings as normal or mildly abnormal. The greater median nerve CSA and higher wrist-to-forearm ratio in wrists with mildly abnormal EDX findings likely reflect more advanced compression leading to intraneural edema and ischemia sufficient to disrupt nerve conduction, while the presence of abnormal CSA in 26% of EDX-normal wrists underscores the utility of US as a complementary diagnostic tool.^36^ Another study provided evidence that the prevalence of underdiagnosed CTS in patients with FM may be higher than previously reported. Therefore, the authors emphasized the importance of ultrasonographic evaluation in symptomatic patients in addition to EDX.^37^ In contrast, no statistically significant difference was detected in median nerve CSA between FM patients and the control group, and these results align with those of Silva et al.^38^ Furthermore, median nerve CSA did not differ significantly between fibromyalgia patients with and without CTS.

A few studies have demonstrated a statistically significant association between FIQ scores in FM patients and CTS severity.^39^^,^^40^ The present study’s results emphasize that the presence of CTS in FM had an impact on symptoms, functionality, and disease severity, as measured by the BCTQs, BCTQf, and FIQ. Ultrasound measurements (CSA, distal/proximal ratio) were also associated with clinical scales (BCTQs, BCTQf, and FIQ).

Patients with FM frequently experience paresthesia in their extremities, presumably due to central sensitization, causing abnormal sensory perception. These individuals exhibit increased subjective sensitivity to multiple sensory stimuli and reduced tolerance for non-nociceptive sensory input, which can complicate the interpretation of paresthetic symptoms in CTS. This consideration is crucial because most hand surgeons rely on patient history and physical examination to manage CTS.^41^ Failure to correctly identify the cause of the symptoms may result in unsatisfactory outcomes from unnecessary carpal tunnel interventions. Studies have demonstrated that FM patients often experience poor results from steroid injections and median nerve release surgery.^42^ Conversely, paresthetic symptoms should alert physicians to assess for potential unidentified CTS in patients with FM, as delayed diagnosis can result in disease progression and irreversible nerve damage. Sonographic evaluation appears to correlate with symptom severity, functional status, FM disease severity, and CTS severity according to EDX in FM patients with CTS. The results of this study indicate the importance of CTS screening in FM. Further research is required to confirm these results.

Studies in rheumatoid arthritis (RA) and psoriatic arthritis (PsA) have shown that CTS in these conditions is frequently associated with synovial proliferation and flexor tendon sheath inflammation, leading to marked enlargement of the median nerve. Hammer et al^43^ reported mean CSA values around 13-14 mm^2^ in RA-related CTS, while recent studies in PsA have demonstrated comparable or slightly higher CSA values, ranging from 13 to 15 mm^2^.^44^^,45^ Smerilli et al^46^ similarly observed increased CSA and hypoechogenicity in RA compared with idiopathic CTS. In contrast, the CSA enlargement observed in the current FM cohort was generally smaller, suggesting a milder degree of structural nerve involvement. This comparison supports the notion that, in FM, peripheral nerve changes may occur without the marked inflammatory or compressive pathology typically seen in inflammatory arthritides.

This study has several strengths. First, a power analysis was conducted to calculate the sample size, and data were collected prospectively. Assessments were performed using valid and reliable clinical scales such as the BCTQs, BCTQf, and FIQ. Physicians who assessed the EDX results and performed the US were blinded to the clinical characteristics of the patients. However, this research has certain limitations that warrant consideration. There was no long-term follow-up of patients with FM. No causal relationship was established due to the cross-sectional study design.

In conclusion, FM and CTS can coexist and/or mimic each other’s symptoms, and CTS is more prevalent in FM patients compared to the general population. It is essential to determine whether the patient’s symptoms are attributable to FM or any associated CTS to avoid unnecessary interventions and provide appropriate treatment. Furthermore, US parameters showed significant associations with the severity of CTS and clinical scale scores in patients with FM.

## Figures and Tables

**Table 1.** Demographic Characteristics of the Participants

	**FM (n = 40)**	**Control (n = 36)**	*P*
Age (years), median (IQR)	43.5 (33.25-52)	36.5 (31-41)	.375*
Body mass index, median (IQR)	26.5 (23.9-28.3)	27.75 (23.97-34.52)	.941*
Occupational risk factors, n (%)	4 (10)	3 (8.3)	>.99**
Right hand dominancy, n (%)	35 (87.5)	32 (88.9)	>.99**

FM, fibromyalgia; IQR, interquartile range.

*Mann–Whitney *U*-test.

**Fisher’s Exact test.

**Table 2.** Clinical Characteristics, Electrodiagnostic Studies Results and Ultrasound Findings

		**FM Wrists**	**Control Wrists**	*P*
Presence of paresthesia, n (%)		50 (62.5)	2 (2.8)	<.001**
Tinel sign (+), n (%)		16 (20.0)	2 (2.8)	<.001**
Phalen sign (+), n (%)		12 (15.0)	0 (0)	<.001**
Sensory deficit, n (%)		22 (27.5)	2 (2.8)	<.001**
Motor deficit, n (%)		4 (5.0)	0 (0)	<.001**
EDX, n (%)	Not required/Normal	62 (77.5)	70 (97.2)	<.001**
	Mild/Moderate CTS	18 (22.5)	2 (2.8)	
US findings	Presence of intraneural PDS, n (%)	12 (15)	4 (5.6)	.058**
	Median nerve CSA (mm^2^), median (IQR)	8 (6.8-9)	8 (6.9-8.9)	.727*
	Distal/proximal ratio, median (IQR)	0.90 (0.80-1.20)	0.98 (0.90-1.05)	.270*

CSA, cross-sectional area; CTS, carpal tunnel syndrome; EDX, electrodiagnostic studies; FM, fibromyalgia; IQR, interquartile range; PDS, power Doppler signal; US, ultrasound.

*Mann–Whitney *U*-test.

**Chi-square test.

**Table 3.** Comparison of Ultrasound Findings and Clinical Scales in Fibromyalgia Groups

		**FM Wrists with CTS** **(n = 18)**	**FM Wrists Without CTS** **(n = 62)**	*P*
US findings	Presence of intraneural PDS, n (%)	8 (44.4)	4 (6.5)	<.001**
	Median nerve CSA (mm^2^), median (IQR)	8.2 (6.8-9.4)	7.1 (6.2-8)	.141*
	Distal/proximal ratio median (IQR)	1.66 (1.44-1.72)	0.85 (0.75-0.97)	.001*
Clinical scales	BCTQs, median (IQR)	33 (30.75-33.25)	17 (14-29)	<.001*
	BCTQf, median (IQR)	24 (16-24)	15 (11-16)	<.001*
	FIQ, median (IQR)	59.88 (53.88-67.55)	48.10 (32.50-58.20)	.001*

BCTQf, Boston Carpal Tunnel Syndrome Questionnaire functional status; BCTQs, Boston Carpal Tunnel Syndrome Questionnaire symptom severity; CSA, cross-sectional area; CTS, carpal tunnel syndrome; FIQ, Fibromyalgia Impact Questionnaire; FM, fibromyalgia; IQR, interquartile range; PDS, power Doppler signal; US, ultrasound.

*Mann–Whitney *U*-test.

**Fisher’s Exact test.

**Table 4.** Correlation Between Ultrasound Findings and Clinical Scales in Fibromyalgia Patients

	**Median Nerve CSA (mm** **^2^)**		**Distal/Proximal Ratio**	
	*r*	P*	*r*	P*
BCTQs	0.557	<.001	0.431	<.001
BCTQf	0.599	<.001	0.435	<.001
FIQ	0.553	<.001	0.336	.002

BCTQf, Boston Carpal Tunnel Syndrome Questionnaire functional status; BCTQs, Boston Carpal Tunnel Syndrome Questionnaire symptom severity; CSA, cross-sectional area; FIQ, Fibromyalgia Impact Questionnaire.

*Spearman test.

## Data Availability

The data that support the findings of this study are available on request from the corresponding author.
